# A Dynamic Path-Planning Method for Obstacle Avoidance Based on the Driving Safety Field

**DOI:** 10.3390/s23229180

**Published:** 2023-11-14

**Authors:** Ke Liu, Honglin Wang, Yao Fu, Guanzheng Wen, Binyu Wang

**Affiliations:** State Key Laboratory of Automotive Simulation and Control, Jilin University, Changchun 130022, China; keliu@jlu.edu.cn (K.L.); whl22@mails.jlu.edu.cn (H.W.); wengz20@mails.jlu.edu.cn (G.W.); bywang18@mails.jlu.edu.cn (B.W.)

**Keywords:** intelligent vehicle, driving safety field, obstacle avoidance, path planning

## Abstract

Establishing an accurate and computationally efficient model for driving risk assessment, considering the influence of vehicle motion state and kinematic characteristics on path planning, is crucial for generating safe, comfortable, and easily trackable obstacle avoidance paths. To address this topic, this paper proposes a novel dual-layered dynamic path-planning method for obstacle avoidance based on the driving safety field (DSF). The contributions of the proposed approach lie in its ability to address the challenges of accurately modeling driving risk, efficient path smoothing and adaptability to vehicle kinematic characteristics, and providing collision-free, curvature-continuous, and adaptable obstacle avoidance paths. In the upper layer, a comprehensive driving safety field is constructed, composed of a potential field generated by static obstacles, a kinetic field generated by dynamic obstacles, a potential field generated by lane boundaries, and a driving field generated by the target position. By analyzing the virtual field forces exerted on the ego vehicle within the comprehensive driving safety field, the resultant force direction is utilized as guidance for the vehicle’s forward motion. This generates an initial obstacle avoidance path that satisfies the vehicle’s kinematic and dynamic constraints. In the lower layer, the problem of path smoothing is transformed into a standard quadratic programming (QP) form. By optimizing discrete waypoints and fitting polynomial curves, a curvature-continuous and smooth path is obtained. Simulation results demonstrate that our proposed path-planning algorithm outperforms the method based on the improved artificial potential field (APF). It not only generates collision-free and curvature-continuous paths but also significantly reduces parameters such as path curvature (reduced by 62.29% to 87.32%), curvature variation rate, and heading angle (reduced by 34.11% to 72.06%). Furthermore, our algorithm dynamically adjusts the starting position of the obstacle avoidance maneuver based on the vehicle’s motion state. As the relative velocity between the ego vehicle and the obstacle vehicle increases, the starting position of the obstacle avoidance path is adjusted accordingly, enabling the proactive avoidance of stationary or moving single and multiple obstacles. The proposed method satisfies the requirements of obstacle avoidance safety, comfort, and stability for intelligent vehicles in complex environments.

## 1. Introduction

The development of intelligent driving technology holds significant importance in enhancing driving safety, improving travel efficiency, alleviating traffic congestion, and enhancing driving comfort. Among the various core technologies of intelligent vehicles, local path planning plays a crucial role in converting sequences of driving behavior decisions into executable local motion paths or trajectories of vehicle controllers. Consequently, local path planning serves as a key factor in determining driving quality and ensuring operational vehicle safety, thereby occupying a vital position in intelligent vehicle research [1]. Path planning for obstacle avoidance for intelligent vehicles refers to the real-time generation of collision-free motion paths from the starting point to the target destination in environments comprising dynamic and static obstacles. This process aims to optimize performance criteria, including operational safety, driving smoothness, and passenger comfort, while simultaneously satisfying constraints related to vehicle kinematics, dynamics, road geometry, and traffic regulations. As such, it involves solving a multi-constrained, multi-objective optimization problem [2].

To generate safe, comfortable, and easily trackable obstacle avoidance paths, the problem of path planning requires the establishment of an accurate and computationally efficient driving risk assessment model. It is essential to consider the influence of the vehicle’s motion state and kinematic characteristics on path planning to enhance the model’s adaptability to dynamic driving scenarios. For the path-planning problem, scholars at home and abroad have carried out extensive research, and the commonly used local path-planning methods can be roughly categorized into five categories: the graph search method, the sampling-based method, the geometric curve method, the artificial potential field method, and the optimal control method [3]. The graph search method utilizes grids or lattices to transform the vehicle’s drivable area into a graph representation. Then, based on certain rules (such as the shortest path or optimal efficiency, etc.), it searches for a path within the graph that meets the specified requirements. Commonly used methods include Dijkstra, A*, D*, etc. [4,5]. However, this method often exhibits curvature-discontinuous paths, making it unsuitable for direct application in vehicle tracking control. It also involves a significant computational burden for online calculations, resulting in poor real-time performance. Moreover, the search performance in high-dimensional spaces tends to degrade significantly. The sampling-based method involves constructing a connected graph by uniformly or randomly sampling the state space. It then determines whether to continue extending and searching for the next sample point when the initial and target states are within the graph or can be connected to the graph. Commonly used methods include probabilistic road map (PRM) and rapidly exploring random tree (RRT) [6,7]. However, using this method often results in curvature-discontinuous paths and has limited convergence in dynamic environments. Random sampling lacks guidance, making it difficult to accurately satisfy complex constraints. The geometric curve method involves using a specific type of curve to generate a smooth path from a series of discrete waypoints via interpolation or fitting. Commonly used curves include polynomial curves, Bézier curves, spline curves, etc. [8,9]. However, using this method, the quality of the planned path is influenced by the choice of waypoints or control points, making it challenging to balance exterior risks and path continuity. Additionally, using specific types of curves to describe the path may restrict the full utilization of the vehicle’s motion capabilities. The artificial potential field method involves establishing a repulsive potential field around obstacles and an attractive potential field around the target point to guide the vehicle toward the goal and accomplish path planning. Various improved path-planning methods based on the APF have been proposed for scenarios such as multi-vehicle interaction, overtaking, lane changing, and others [10,11,12]. However, this method has several drawbacks, including the possibility of unreachable target points and susceptibility to local minima. It also exhibits poor adaptability to dynamic scenarios. Furthermore, improving the potential field function requires the calibration of numerous parameters, making the model parameter calibration process cumbersome. The optimal control method involves solving an optimization problem based on predefined objectives and constraints to generate an optimal path within the drivable region. Commonly used methods include model predictive control (MPC) [13,14]. However, directly solving the nonlinear optimal control problem that accurately describes vehicle motion planning propositions can be challenging. It often requires significant computational resources and involves the linearization or convexification of the original model. Among them, the artificial potential field method has the advantages of a simple mathematical model and small online computation, which is widely used to solve local path-planning problems. In practical applications, using a certain motion planning method independently to solve the obstacle avoidance motion planning problem in complex scenes is uncommon, and it is often used to complete the path-planning task through the combination of various methods, with the help of the advantages of two or more methods. For example, the algorithm employed in [15,16] utilizes a combination of geometric curves and an optimal approach. The authors of [17] incorporated spatial sampling and geometric curve interpolation methods. The authors of [18] integrated spatial sampling with optimal control methods. The authors of [19,20] employed a combination of artificial potential fields and optimal control to achieve continuous path planning for mobile robots in narrow spaces.

In 1986, Khatib proposed the artificial potential field algorithm and introduced it into the field of robot path planning [21]. However, this method has some problems, such as unreachable target position, local extreme value, failure to consider obstacle motion state and vehicle motion constraint, etc., which greatly reduces the safety of intelligent vehicles using the artificial potential field method in the actual obstacle avoidance process and the success rate of path planning for obstacle avoidance [22]. To address these problems, the authors of [23] considered obstacle constraints and vehicle constraints and used the Gaussian combined affiliation function to establish the target gravitational point function, which solves the problem of local minima in the traditional artificial potential field method. The authors of [24] constructed the obstacle point model with the minimum safe distance as the symmetric distance, which ensures that the local target position is on the symmetry axis line and solves the problem of target unreachability and local minima. In [25] and other studies, trigonometric functions were used to construct the road boundary potential field, and exponential functions were used to construct the obstacle potential field, which can effectively plan a safe obstacle avoidance path in the face of the existence of obstacles in the scene. The authors of [26] used an artificial potential field for steering obstacle avoidance path planning in the presence of dynamic obstacle scenarios, establishing an elliptically distributed obstacle potential field with the long axis of the ellipse varying with the relative position, which allows for the online planning of collision avoidance paths based on the predicted motion position of pedestrians. However, these studies did not consider factors such as vehicle attributes, the relative motion state between the driving vehicle and the obstacle, and road conditions when constructing the artificial potential field, and the swerving obstacle avoidance paths are different when the relative velocity of the driving vehicle and the obstacle are different. The vehicle path planning for the obstacle avoidance process needs to consider the comprehensive impact of people, vehicles, roads, and other traffic elements on the obstacle avoidance path planning to ensure that the driving vehicle has a sufficient longitudinal and transverse safety distance to complete the obstacle avoidance task.

Considering the similar characteristics between the driving risks posed by traffic factors to driving vehicles and the fields in physics, Wang et al. proposed the concept of the “driving safety field” to quantitatively evaluate the risks posed by various elements in traffic to vehicles on the road [27,28]. Objects in the traffic scene that can cause driving risks can be primarily classified into three categories: “human” (the driver), “vehicle” (moving objects on the road), and “road” (static elements in the road environment). On the road, all objects that pose risks to driving vehicles form a driving safety field, which repels other objects that approach it. Accordingly, the driving safety fields formed by these three categories of objects are defined as the “behavior field”, “kinetic field”, and “potential field”, respectively. Building upon this concept, this paper proposes a dynamic obstacle avoidance path-planning method based on the driving safety field. By constructing a comprehensive driving safety field composed of the potential field generated by static obstacles, the kinetic field generated by dynamic obstacles, the potential field generated by lane boundaries, and the driving field generated by the target position, a virtual mechanical system for vehicle motion is established. Through force analysis, the resultant force direction of the virtual field forces is used to guide the vehicle’s forward motion, generating a motion path that satisfies the vehicle’s kinematic and dynamic constraints while maintaining a curvature-continuous path. As the comprehensive driving safety field model considers the motion states of the ego vehicle and other traffic participants, the proposed dynamic path-planning method for obstacle avoidance can automatically adjust the starting position of obstacle avoidance behavior based on the motion states of the ego vehicle and the obstacle vehicle. The higher the relative velocity between the ego vehicle and the obstacle vehicle, the farther the starting position of the obstacle avoidance path is from the obstacle vehicle. This enables the active avoidance of stationary or moving single and multiple obstacles, and meeting the safety, comfort, and stability requirements of intelligent vehicles in complex environments.

## 2. A Path-Planning Framework Based on the Driving Safety Field

**Figure 1:**
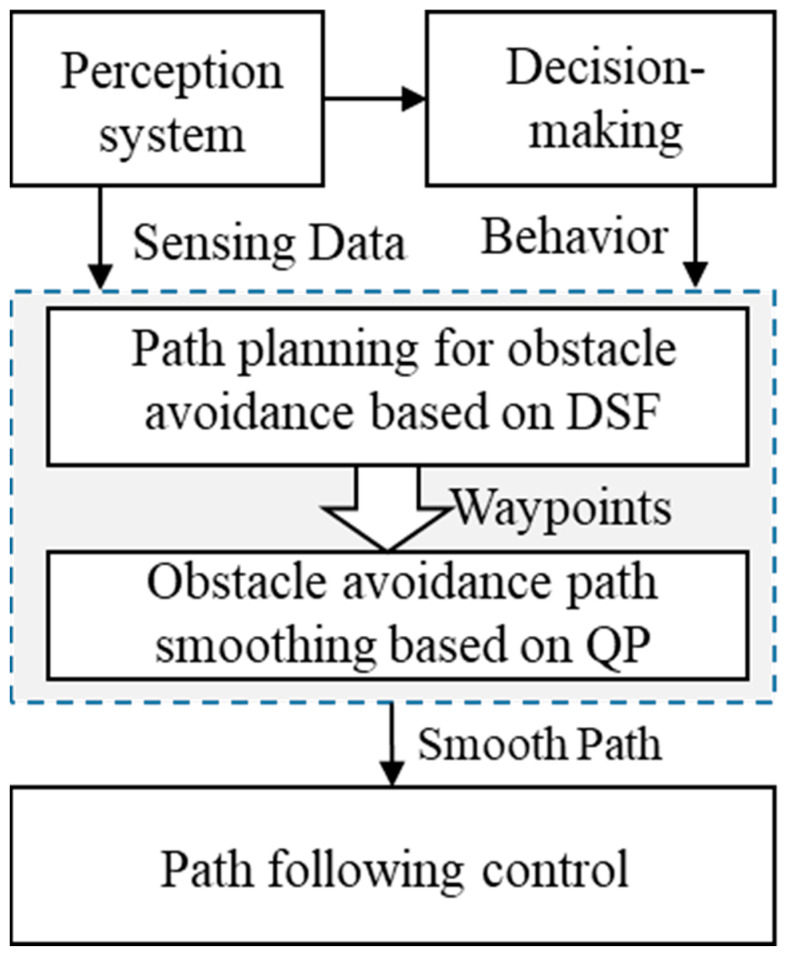
Path-planning framework based on the DSF.

In order to devise an obstacle avoidance path that ensures safety, smoothness, and compliance with the vehicle’s motion characteristics, this paper introduces a dynamic obstacle avoidance path-planning method with a two-layered structure, as depicted in Figure 1. Leveraging this two-layered structure, obstacle avoidance path planning can be divided into two distinct steps. The first step encompasses the initial path planning, which incorporates the results from the perception system and behavior decision making as inputs. By comprehensively considering the vehicle’s kinematic and dynamic constraints, along with the collision constraints, a collision-free path is planned from the current position to the target position, effectively satisfying the vehicle’s motion characteristics. Subsequently, the second step entails path smoothing for obstacle avoidance. Utilizing the previously planned path as the initial solution, a quadratic programming method is employed to optimize and generate a curvature-continuous and smooth obstacle avoidance path. This optimized path is then transferred as the control target to the path-tracking module.

**Figure 2:**
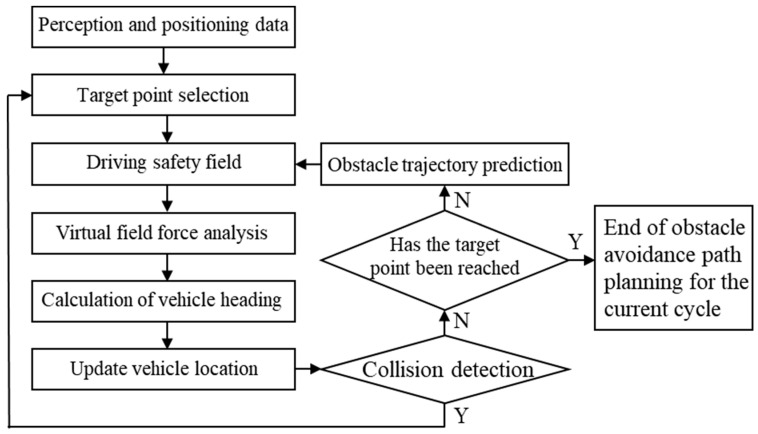
Dynamic path-planning method for obstacle avoidance based on the DSF.

The initial path-planning process for dynamic obstacle avoidance, based on the driving safety field, is illustrated in Figure 2, corresponding to Step 1 in Figure 1. During the initiation of obstacle avoidance path planning, the vehicle’s driving behavior (e.g., lane changing, stopping, following, etc.) is determined based on perception and localization information. The target position area is selected based on the driving behavior, the motion states of the ego vehicle and surrounding vehicles in the target lane at the start of obstacle avoidance, and the road regulations. Typically, multiple points are selected within this area using equidistant intervals, and these points are usually located on the centerline of the target lane. In this paper, we adopt the method proposed in [29] to calculate the endpoint area for obstacle avoidance. The center point of this area is selected as the target position point for obstacle avoidance. Using the theory of the driving safety field, a comprehensive driving safety field model is established for obstacle avoidance scenarios by incorporating driving scene perception information and the target position. The virtual field forces acting on the ego vehicle within the comprehensive driving safety field are computed, and force analysis is performed. By considering both the magnitude and direction of the resultant force experienced by the ego vehicle in the comprehensive driving safety field, the vehicle’s target heading angle is calculated, considering the vehicle’s kinematic and dynamic constraints. If the difference between the target heading angle and the current heading angle exceeds the threshold determined by the steering system, the maximum steering angle of the vehicle is selected as the target steering angle for the next step. The next-step position is then updated accordingly, utilizing the principles of vehicle kinematics. At this point, collision detection is performed to ensure the safety of the planned avoidance path. If the collision constraint is satisfied, the next step is executed. If the collision constraint is not met, a new target position point is selected, and a new comprehensive driving safety field is constructed. The path-planning process is restarted from the current position of the ego vehicle. For example, if a sudden obstacle (such as a vehicle braking abruptly) appears on the planned path, then when calculating the next-step target heading angle for the ego vehicle based on the comprehensive driving risk field, it is determined that even with the maximum steering angle, the vehicle cannot avoid a collision with the sudden obstacle. In such a situation, the path planner needs to reassess and generate a feasible path to avoid multiple obstacles. If the difference between the target heading angle and the current heading angle is smaller than the predetermined threshold set by the steering system, collision detection is unnecessary. Instead, it is directly determined whether the vehicle has reached the target position. If the target position has not been reached, obstacle trajectory prediction is performed to update the comprehensive driving safety field model. Based on the updated safety field model, the aforementioned steps are repeated to further update the next-step position. Once the target position is reached, the obstacle avoidance path planning for the current cycle is completed.

## 3. Obstacle Avoidance Path-Planning Method for Dynamic Obstacles

### 3.1. Coordinate System Definition

**Figure 3:**
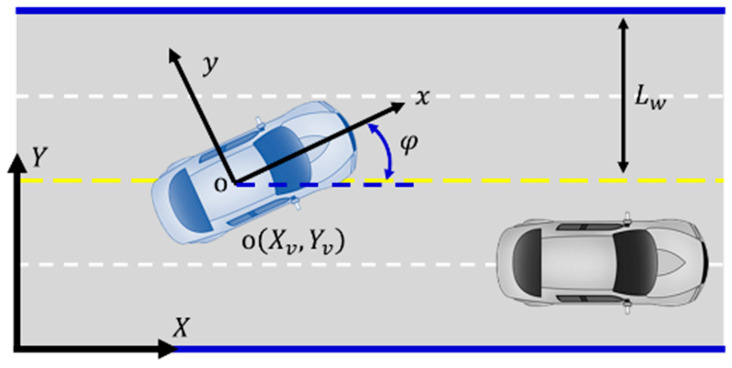
Coordinate system definition.

The vehicle body and global coordinate systems are defined in Figure 3. The vehicle body coordinate system (xoy) has its origin at the vehicle’s center of mass and follows the right-hand coordinate system convention. The X-axis is defined as the positive direction along the vehicle’s centerline, extending forward. The Y-axis is defined as the positive direction perpendicular to the vehicle’s centerline, pointing to the right. The global coordinate system (XOY) is defined with an arbitrary point before any vehicle’s movement in the driving scene as the origin. It also adheres to the right-hand coordinate system convention. The X-axis is defined as the positive direction along the road’s centerline in the direction of the vehicle’s motion. The Y-axis is defined as the positive direction perpendicular to the X-axis, pointing toward the left boundary of the road. The coordinate systems are measured in meters (m) unless otherwise specified.

### 3.2. Driving Safety Field Modeling

#### 3.2.1. Lane Line Potential Field

According to the theory of the driving safety field, the lane line belongs to the second category of stationary objects. The field strength, denoted as $E_{R,aj}$, of the potential field formed by lane line markings “*a*” at position $\left(X_{j},Y_{j}\right)$ is expressed as follows:

(1) $$E_{R,aj}={LT}_{a}\cdot R_{a}\cdot {\left(\frac{L_{w}}{2}-\left|r_{aj}\right|\right)}^{k_{1}}\cdot \frac{r_{aj}}{\left|r_{aj}\right|}$$

where ${LT}_{a}$ denotes the type of lane line, $L_{w}$ is the lane width, $r_{aj}=\left(X_{j}-X_{a},Y_{j}-Y_{a}\right)$ is the distance vector from lane line a to any point $\left(X_{j},Y_{j}\right)$ within the lane, and $\left|r_{aj}\right|$ ranges over $\left[0,L_{w}/2\right]$. The coordinates $\left(X_{a},Y_{a}\right)$ represent the projection point of the point $\left(X_{j},Y_{j}\right)$ onto lane line a. $R_{a}$ represents the road condition factor at the point $\left(X_{a},Y_{a}\right)$. The direction of $E_{R,aj}$ is the same as that of $r_{aj}$, which is the direction of the gradient descent of the field strength. Furthermore, $E_{R,aj}$ increases as $\left|r_{aj}\right|$ decreases. Additionally, $k_{1}$ is a positive constant that needs to be determined.

When there are obstacles or slower vehicles on the road, the ego vehicle typically chooses to change lanes for obstacle avoidance. In this situation, the potential field generated by the road lane line between the current lane and the target lane will no longer constrain the vehicle. Instead, the potential field created by the lane line will be influenced by the position of the obstacles.

(2) $$E_{R}\left(X,Y\right)=E_{R,aj}\times A\left(Y\right)$$

**Figure 4:**
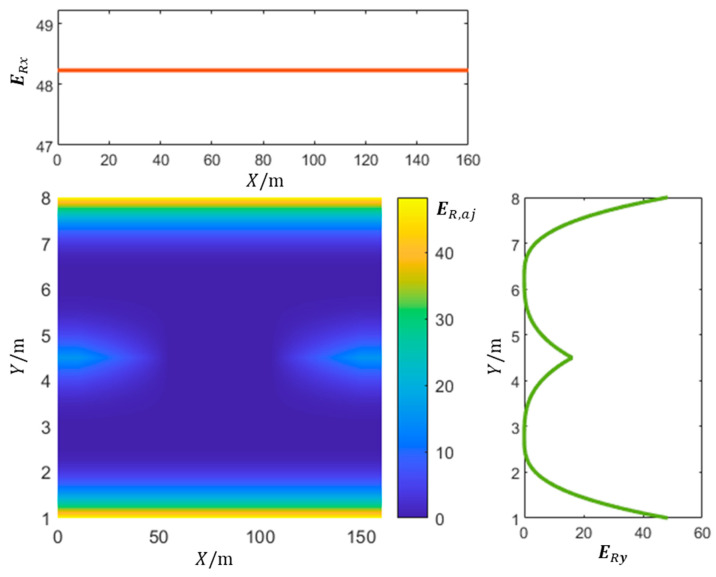
Distribution of potential field strength generated by lane lines.

In the equation above, the function $A\left(Y\right)$ serves the purpose of generating a low potential field region across the ego lane and the target lane when there is an obstacle ahead of the ego vehicle. This facilitates the lane-changing maneuver to overtake the obstacle and avoid a collision. The calculation formula for $A\left(Y\right)$ is as follows:

(3) $$A\left(Y\right)=\left\{\begin{array}{cc} 0 & \left|\left.Y-Y_{0}\right|{\leq D}_{b}\right.\\ \frac{\left|\left.Y-Y_{0}\right|{-D}_{b}\right.}{D_{t}{-D}_{b}} & D_{b}\leq \left|\left.Y-Y_{0}\right|{\leq D}_{t}\right.\\ 1 & else \end{array}\right.$$

where the coordinates of the obstacle’s center are represented by $\left(X_{0},Y_{0}\right)$. The maximum effective range of the potential field created by the obstacle ahead of the ego vehicle is denoted as $D_{t}$, whereas $D_{b}$ corresponds to the minimum braking distance of the ego vehicle. Figure 4 shows the distribution of the potential field strength generated by lane line obstacles or slower vehicles on the road. In the global coordinate system (XOY) shown in Figure 3, $E_{Rx}$ represents the variation in the lane line potential field strength along the X-axis, while $E_{Ry}$ represents the variation in the lane line potential field along the Y-axis.

#### 3.2.2. Static Obstacle Potential Field

**Figure 5:**
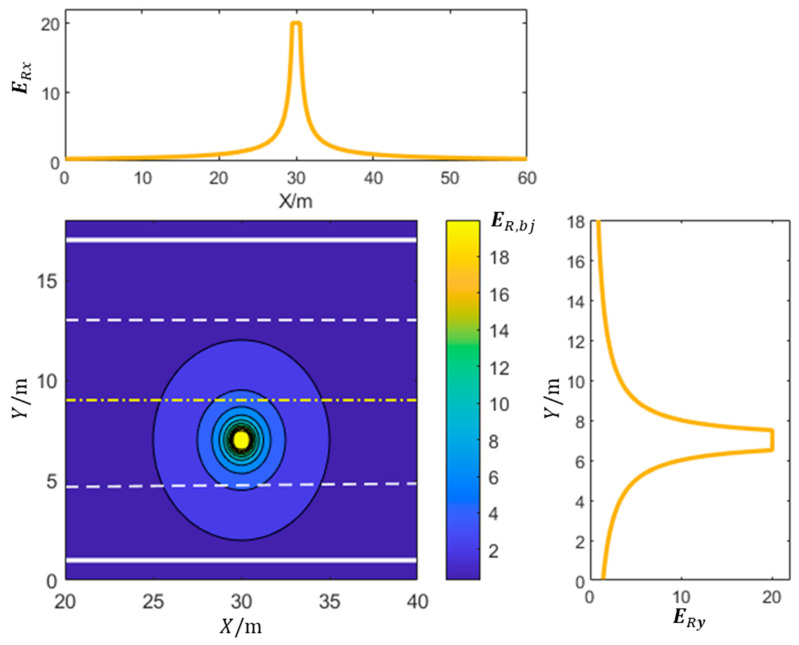
Distribution of potential field strength generated by static obstacles.

Static obstacles on the road mainly include stationary vehicles, guardrails, roadblocks, and so on. Based on the theory of the driving safety field, static obstacles are classified as the first category of stationary objects. The potential field generated by static obstacle “b” at position $\left(X_{j},Y_{j}\right)$ is calculated as the field strength $E_{R,bj}$ and can be expressed with the following equation:

(4) $$E_{R,bj}=\frac{K\cdot R_{b}{\cdot M}_{b}}{{\left|r_{bj}\right|}^{k_{1}}}\cdot \frac{r_{bj}}{\left|r_{bj}\right|}$$

where the centroid position of object *b* is denoted as $\left(X_{b},Y_{b}\right)$, and $r_{bj}=\left(X_{j}-X_{b},Y_{j}-Y_{b}\right)$ represents the distance vector from the centroid of object *b* to the point $\left(X_{j},Y_{j}\right)$. $M_{b}$ denotes the virtual mass of object *b*, $R_{b}$ represents the road condition factor at point $\left(X_{b},Y_{b}\right)$, and K(>0) and $k_{1}(>1)$ are constants to be determined. Figure 5 shows the distribution of the potential field strength generated by static obstacles. It should be noted that the maximum constant values for $E_{Rx}$ and $E_{Ry}$ in Figure 5 are not derived directly from Equation (4). The purpose of these figures is to provide a visual representation of the potential field and their variation along the respective axes. The maximum constant values for $E_{Rx}$ and $E_{Ry}$ are determined based on the sizes of static obstacles and the minimum safety distance between the ego vehicle and these obstacles. These values are chosen to ensure a suitable range of potential field strength values that effectively guide the path-planning process while considering driving safety and obstacle avoidance.

#### 3.2.3. Dynamic Obstacle Kinetic Field

**Figure 6:**
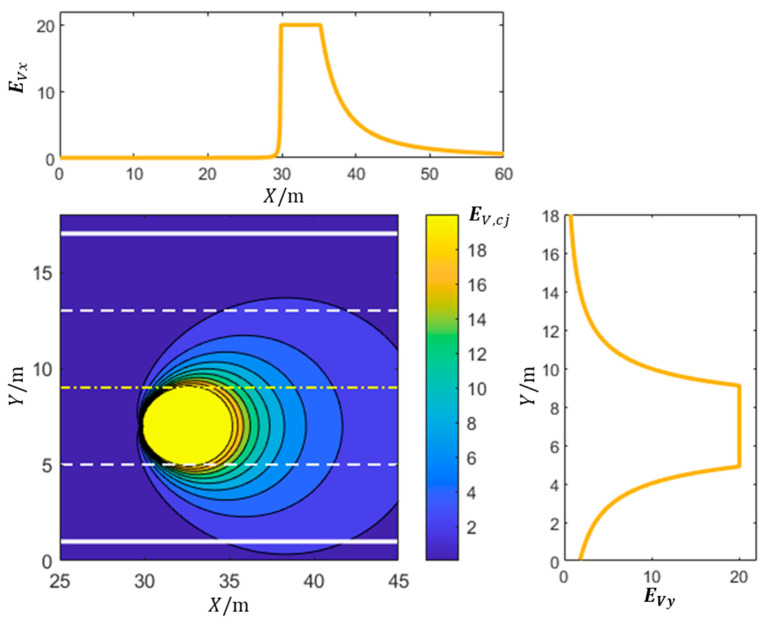
Distribution of potential field strength generated by dynamic obstacles.

The kinetic field generated by dynamic obstacles is influenced by the characteristics of the objects (type, mass, and velocity), their motion state, and road conditions. The field strength, denoted as $E_{V,cj}$, of the kinetic field created by moving object “*c*” at position $\left(X_{c},Y_{c}\right)$ at point $\left(X_{j},Y_{j}\right)$ can be calculated using the following equation:

(5) $$E_{V,cj}=\frac{K\cdot R_{c}\cdot M_{c}}{{\left|r_{cj}\right|}^{k_{2}}}\frac{r_{cj}}{\left|r_{cj}\right|}exp\left(k_{3}\cdot v_{c}\cdot \cos\left(\theta_{c}\right)\right)$$

where the virtual mass of object “*c*” is denoted as $M_{c}$, while $R_{c}$ signifies the road condition influence factor at $\left(X_{c},Y_{c}\right)$. The distance vector from the center of mass of object “*c*” to the coordinates $\left(X_{j},Y_{j}\right)$ is defined as $r_{cj}=\left(X_{j}-X_{c},X_{j}-Y_{c}\right)$. The velocity vector of object “*c*” is represented by $v_{c}$, and $\theta_{c}$ denotes the angle between $r_{cj}$ and $v_{c}$. Constants K(>0), $k_{2}(>1)$, and $k_{3}(>0)$ are undetermined positive constants. Figure 6 shows the distribution of potential field strength generated by dynamic obstacles. It should be noted that the maximum constant values for $E_{Vx}$ and $E_{Vy}$ are not derived directly from Equation (5). The purpose of these figures is to provide a visual representation of the kinetic fields and their variation along the respective axes. The maximum constant values for $E_{Vx}$ and $E_{Vy}$ are determined based on the sizes and motion state of dynamic obstacles and the minimum safety distance between the ego vehicle and these obstacles. These values are chosen to ensure a suitable range of kinetic field strength values that effectively guide the path-planning process while considering driving safety and obstacle avoidance.

#### 3.2.4. Target Position Driving Field

**Figure 7:**
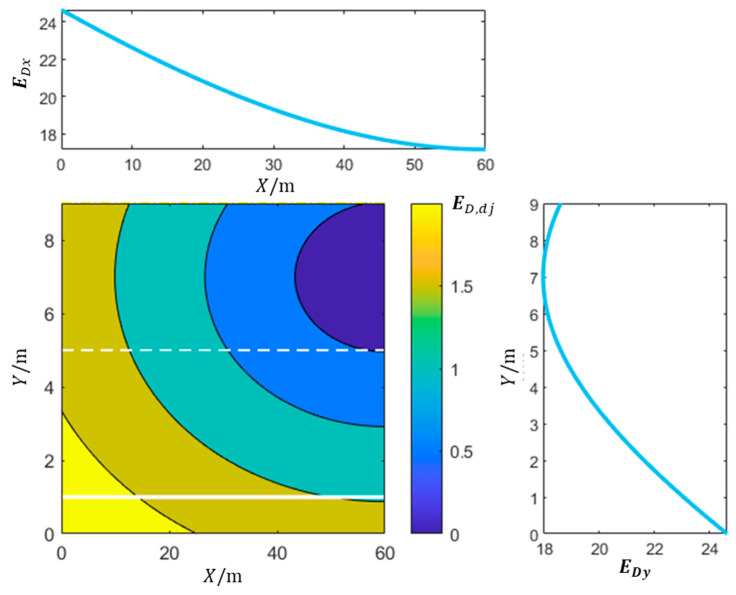
Distribution of the driving field strength generated by the target position.

The driving field is derived from the ego vehicle’s need to efficiently and safely complete driving tasks, enabling it to reach the target destination. The vehicle’s powertrain system generates driving force, while the steering system produces the steering angle to propel the vehicle forward. The driving field is influenced by various factors, including the ego vehicle’s attributes (type and position), the target location (relevant to driving behavior), the motion state (vehicle velocity), and road conditions. The field strength vector at point $\left(X_{j},Y_{j}\right)$, denoted as $E_{D,dj}$, generated by the target position “*d*” at coordinates $\left(X_{d},Y_{d}\right)$ is expressed with the following equation:

(6) $$E_{D,dj}=k_{D}R_{d}g\sqrt{1-{cos}^{2}\left(\theta_{i,x}\right){cos}^{2}\left(\theta_{i,y}\right)}\frac{r_{dj}}{\left|r_{dj}\right|}$$

(7) $$\theta_{i,x}=\arcsin\left(k_{if}\frac{v_{d}}{v_{l}}\frac{\left|X_{d}-X_{j}\right|}{\left|X_{d}-X_{v}\right|}\right)$$

(8) $$\theta_{i,y}=\arcsin\left(k_{ic}\frac{\left|Y_{d}-Y_{j}\right|}{L_{w}}\right)$$

where $v_{d}$ represents the ego vehicle’s velocity, $v_{l}$ denotes the maximum speed limit on the current lane, and g represents the acceleration due to gravity. $X_{v}$ corresponds to the current position of the ego vehicle along the vertical axis. $L_{w}$ represents the lane width, while $k_{D}$, $k_{if}$, and $k_{ic}$ are undetermined positive constants. Figure 7 shows the distribution of the driving field strength generated by the target position.

### 3.3. Path-Planning Method

**Figure 8:**
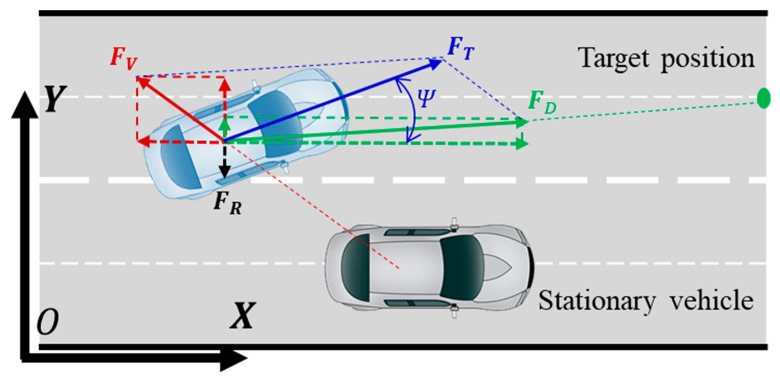
Virtual force analysis of obstacle avoidance path-planning process.

In the path-planning process, the vehicle is simplified as a particle, and the motion state of the vehicle during the planning process is described using parameters such as the position of the vehicle’s center of mass, velocity vector, and heading angle. If we disregard the influence of the lateral deviation angle of the center of mass, the velocity direction and heading angle of the vehicle’s center of mass remain consistent. Figure 8 illustrates a force diagram at the vehicle’s center of mass during the obstacle avoidance path-planning process. $F_{V}$ represents the force exerted on the vehicle by the potential field formed by a stationary obstacle vehicle in the original lane. The force direction follows the line connecting the centers of the two vehicles and moves away from the obstacle vehicle, indicating a repulsive force. $F_{R}$ represents the force exerted on the vehicle by the potential field formed by the lane lines of the target lane. The force direction is perpendicular to the lane lines, constraining the vehicle to travel along the road’s centerline, and also acting as a repulsive force. $F_{D}$ represents the force exerted on the vehicle by the kinetic field formed by the target position in the lane-changing obstacle avoidance process. The force direction follows the line connecting the vehicle’s center of mass and the target position, pointing toward the target position and acting as an attractive force, driving the vehicle toward the target position. The resultant force of the three types of driving safety field forces is denoted as $F_{T}$, and the angle between $F_{T}$ and the road’s centerline is the vehicle’s heading angle, Ψ. As the vehicle’s motion state changes, the direction of the resultant force at the center of mass also changes, and the vehicle moves toward the target position under the driving force of the combined driving safety field, ultimately completing the obstacle avoidance path-planning process. The calculation formula for the driving safety field at the vehicle’s center of mass is as follows:

(9) $$F=E\cdot M_{j}\cdot R_{j}$$

where F represents the force exerted on vehicle j in the driving safety field with the field strength E. $M_{j}$ signifies the equivalent mass of the ego vehicle, and $R_{j}$ denotes the factor accounting for the influence of road conditions at the ego vehicle’s current position.

The combined effect of forces in the driving safety field modifies the magnitude of the vehicle’s center of mass velocity and determines its heading for the subsequent stage. Assuming a lane-changing obstacle avoidance process, the longitudinal component of the center of mass velocity, $V_{x}$, remains constant. Considering the influence of the driving safety field forces, the lateral component of the center of mass velocity, $V_{y}$, can be computed using the following formula based on the heading angle:

(10) $$V_{y}\left(k\right)=V_{x}\left(k\right)\tan\psi \left(k\right)$$

In the obstacle avoidance path-planning process, the variable “*k*” represents the *k*-th stage, and *ψ*(*k*) signifies the heading angle of the vehicle’s center of mass, indicating the direction of the resultant force acting on the center of mass due to the driving safety field.

To update the vehicle’s position for the next stage, a fixed time interval ΔT is employed. The position of the vehicle $\left(X_{v}\left(k+1\right),Y_{v}\left(k+1\right)\right)$ in the (*k* + 1)-th stage is estimated based on the position $\left(X_{v}\left(k\right),Y_{v}\left(k\right)\right)$ and the velocity of its center of mass in the *k*-th stage using the following formula:

(11) $$\left\{\begin{array}{c} X_{v}\left(k+1\right)=X_{v}\left(k\right)+V_{x}\left(k\right)\times \Delta T\\ Y_{v}\left(k+1\right)=Y_{v}\left(k\right)+V_{y}\left(k\right)\times \Delta T \end{array}\right.$$

### 3.4. Vehicle Motion Constraints

The process of lane-changing obstacle avoidance entails not only the provision of collision-free paths to ensure safe passage but also the consideration of the vehicle’s unique kinematic and dynamic characteristics. This guarantees that the vehicle can travel smoothly along the planned path, adhering to the specified time constraints.

#### 3.4.1. Kinematic Constraints

The planned lane-changing obstacle avoidance trajectory requires a continuous curvature, and at the same time, is subject to the constraints of the vehicle’s minimum steering radius $R_{min}$; thus, it must be ensured that the curvature $\kappa \left(k\right)$ of any road point on the planned path is smaller than the corresponding curvature of the vehicle’s minimum steering radius.

(12) $$\kappa \left(k\right)\leq \frac{1}{R_{min}}$$

The obstacle avoidance path is the target of the vehicle tracking control. If the vehicle can accurately track the target path, according to the principle of Ackermann’s steering geometry, from the vehicle kinematics equations, the target steering angle $\delta \left(k\right)$ can be obtained based on the calculation of the planned path curvature:

(13) $$\delta \left(k\right)=arctan\left[\kappa \left(k\right)\times L\right]$$

where L is the wheelbase of the vehicle, measured in meters.

#### 3.4.2. Dynamical Constraints

Dynamic constraints refer to the limitations and restrictions imposed on the vehicle’s motion and behavior due to its physical and mechanical properties. To prevent lateral skidding of the vehicle during the steering process for lane-changing obstacle avoidance and improve the comfort of path-tracking control, certain constraints need to be satisfied for the lateral acceleration $a_{y}\left(k\right)$ and yaw rate $\omega_{r}\left(k\right)$ of the vehicle along the planned path during the lane-changing obstacle avoidance process.

(14) $$\begin{array}{c} \left|a_{y}\left(k\right)\right|\leq a_{y\_max}\\ \left|\omega_{r}\left(k\right)\right|\leq \omega_{max} \end{array}$$

where $a_{y\_max}$ represents the maximum lateral acceleration, measured in m/s^2^; $\omega_{max}$ represents the maximum yaw rate, measured in rad/s. To ensure driving stability during the obstacle avoidance process, the steering operation requires that the lateral acceleration does not exceed 0.4 g, where g represents the gravitational acceleration and is measured in m/s^2^. The steering sensitivity is a commonly employed metric for assessing the precision and stability of the vehicle’s driving direction. The calculation formula is as follows:

(15) $$\frac{\omega_{r}}{\delta }=\frac{u/L}{1+Hu^{2}}$$

where u represents the overall vehicle velocity, measured in m/s; δ denotes the steering angle of the front wheels, measured in rad; and H represents the stability coefficient, measured in s^2^/m^2^. When H=0, the vehicle exhibits neutral steering characteristics. Under this condition, the steering sensitivity is linearly proportional to the vehicle’s speed, and the turning radius remains unaffected by the vehicle’s travel velocity. This characteristic ensures the safety of the vehicle during operation.

(16) $$\delta =\frac{L}{R}$$

Assuming that the lane-changing obstacle avoidance process of the vehicle corresponds to steady-state steering and the lateral deviation angle of the vehicle’s center of mass is minimal, we can approximate the center of mass velocity as the velocity component of the vehicle along the X-axis under constant speed conditions. Consequently, we can determine the lateral acceleration as follows:

(17) $$a_{y}=\frac{u^{2}}{L}\times \delta \leq 0.4g$$

Based on the analysis above, the front-wheel steering angle of the vehicle is affected by the vehicle velocity u, the turning radius $R_{min}$, and the saturation limit of the steering mechanism $\delta_{max}$. Thus, to ensure a smooth lane-changing obstacle avoidance maneuver, the curvature of the planned path must adhere to the following constraints:

(18) $$\kappa \left(k\right)\leq \frac{1}{L}\tan\left(min\left(0.4g\times \frac{L}{u^{2}},\frac{L}{R_{min}},\delta_{max}\right)\right)$$

### 3.5. Vehicle Collision Detection

**Figure 9:**
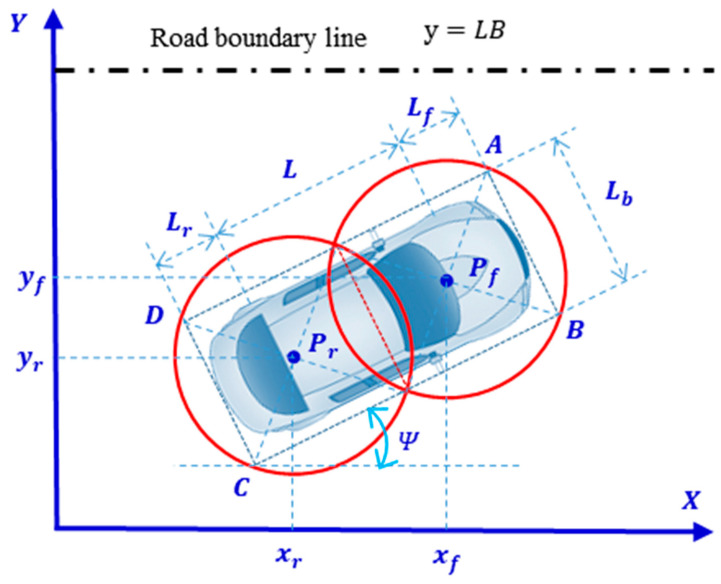
Schematic diagram of collision detection based on double circles describing the vehicle contour.

Two circles with the same radius are used to cover the minimum outer rectangle of the vehicle contour uniformly to realize the approximate description of the vehicle shape in [3], as shown in Figure 9. In the figure, the rectangle ABCD is the minimum outer rectangle of the vehicle contour, the points $P_{f}$ and $P_{r}$ are the centers of the two circles that uniformly cover the vehicle contour, and $R_{c}$ is the radius of the circle that uniformly covers the vehicle contour. According to the geometric relationship, the coordinates of the center of the circle and the radius are calculated as follows:

(19) $$R_{c}=\frac{1}{2}\sqrt{{\left(\frac{L_{r}+L+L_{f}}{2}\right)}^{2}+L_{b}^{2}}$$

(20) $$\begin{array}{c} x_{r}=x_{v}+\frac{1}{4}\left(L_{f}+L-3L_{r}\right)\cos\Psi \\ y_{r}=y_{v}+\frac{1}{4}\left(L_{f}+L-3L_{r}\right)\sin\Psi \\ x_{f}=x_{v}+\frac{1}{4}\left(3L_{f}+L-L_{r}\right)\cos\Psi \\ y_{f}=y_{v}+\frac{1}{4}\left(3L_{f}+L-L_{r}\right)\sin\Psi \end{array}$$

where $L_{f}$ is the distance from the front axle to the forefront of the vehicle; $L_{r}$ is the distance from the rear axle to the rear end of the vehicle; L is the wheelbase; $L_{b}$ is the width of the vehicle; $x_{f}$ and $y_{f}$ are the coordinates of point $P_{f}$; $x_{r}$ and $y_{r}$ are the coordinates of the point $P_{r}$; $x_{v}$ and $y_{v}$ are the coordinates of the center point of the vehicle’s rear axle; and Ψ is the vehicle’s heading angle.

Collision detection between two vehicles based on double circles describing the vehicle contour can then be simplified to determine whether there is an overlapping region between the two circles covering the ego vehicle and any circle covering other vehicles around it. Whether there is an overlap between the two circles can be judged based on the distance between the centers of the two circles; therefore, collision detection between vehicles can be carried out based on the following equation:

(21) $$\left\{\begin{array}{l} dis\left(P_{r\_i},P_{r\_j}\right)\geq R_{c\_i}+R_{c\_j}\\ dis\left(P_{r\_i},P_{f\_j}\right)\geq R_{c\_i}+R_{c\_j}\\ dis\left(P_{f\_i},P_{r\_j}\right)\geq R_{c\_i}+R_{c\_j}\\ dis\left(P_{f\_i},P_{f\_j}\right)\geq R_{c\_i}+R_{c\_j} \end{array}\right.$$

where $P_{r\_i}$, $P_{f\_i}$, $P_{r\_j}$, and $P_{r\_j}$ are the centers of the four circles covering vehicle i and vehicle j, respectively; $R_{c\_i}$ and $R_{c\_j}$ represents the radius of the circles covering vehicle *i* and vehicle *j*, respectively; and $dis\left(P_{r\_i},P_{r\_j}\right)$ is the distance between the two centers.

In addition, it is also necessary to ensure that the vehicle always stays within the road boundary while driving along the planned waypoint, which can be judged by whether the two circles covering the vehicle intersect with the lane boundary. In the scenario shown in Figure 8, the conditions necessary to determine that the vehicle driving along the planned path does not exceed the left road boundary can be expressed as follows:

(22) $$\begin{array}{c} y_{f}\left(k\right)+R_{c}<LB\\ y_{r}\left(k\right)+R_{c}<LB \end{array}$$

where LB is the coordinate value of the road boundary.

## 4. A Path-Smoothing Method Based on Quadratic Programming

### 4.1. Discrete Path Point Smoothing

**Figure 10:**
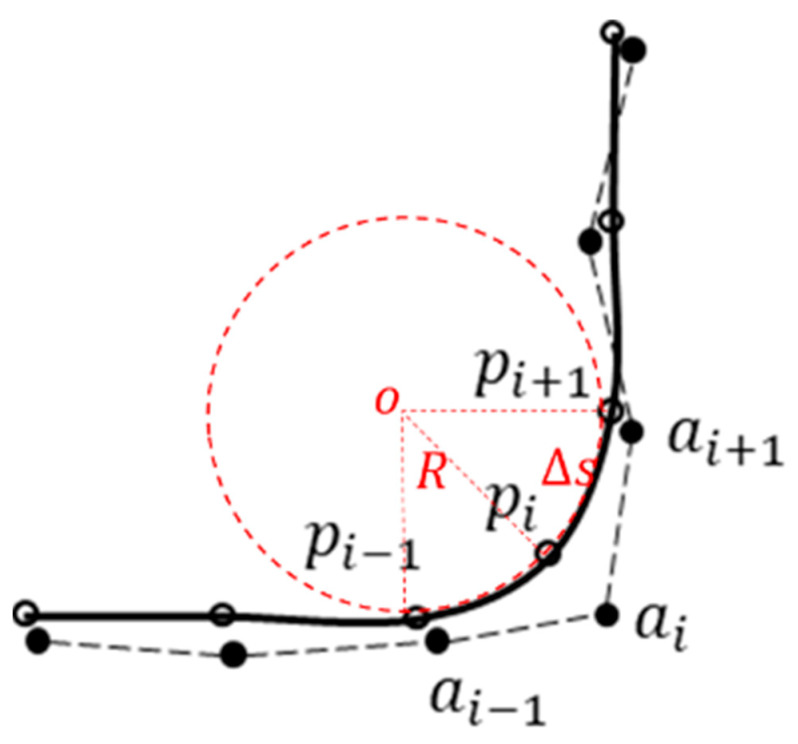
The correspondence between the waypoints of the obstacle avoidance path before and after smoothing.

Let $A_{T}$ represent the set of waypoints in the original obstacle avoidance path planned with the DSF, which is denoted as $A_{T}=\left\{a_{0},a_{1},a_{2},\cdots ,a_{N-1},a_{N}\right\}$. After the smoothing process, the corresponding set of waypoints for the obstacle avoidance path is denoted as $P_{T}=\left\{p_{0},p_{1},p_{2},\cdots ,p_{N-1},p_{N}\right\}$. Here, $a_{i}\left(x_{o\_i},y_{o\_i}\right)$ and $p_{i}\left(x_{i},y_{i}\right)$ represent the coordinates of the waypoints before and after smoothing, respectively. The relationship between the two is illustrated in Figure 10.

#### 4.1.1. Objective Function

Drawing inspiration from the path-smoothing methods described in [30,31], this paper formulates the objective function for the problem of discrete waypoint smoothing, considering both path smoothness and similarity to the original path.

The smoothness of the resulting path increases as the angle between the two-line segments formed by three consecutive waypoints in the smoothed waypoint set becomes larger. This indicates a flatter and smoother path. Therefore, the definition of the objective function for path smoothness is as follows:

(23) $$f_{1}=\sum_{i=1}^{N-1}{\left|\vec{p_{i}p_{i-1}}-\vec{p_{i+1}p_{i}}\right|}^{2}$$

To ensure the similarity in geometry between the smoothed discrete waypoints and the path geometry planned with the DSF, the cost function for geometric similarity is defined as follows:

(24) $$f_{2}=\sum_{i=0}^{N}{\left|\Delta p_{i}a_{i}\right|}^{2}$$

The integrated objective function for waypoint smoothing is defined as follows:

(25) $$Cost=w_{1}f_{1}+w_{2}f_{2}$$

where $w_{1}$ and $w_{2}$ are the weight coefficients of each sub-objective function. If path smoothness is more important than path similarity, $w_{1}$ is set to a larger value than $w_{2}$. On the other hand, if path similarity is more important than path smoothness, $w_{2}$ is set to a larger value than $w_{1}$. In this paper, we set the value of $w_{1}$ to 5 and the value of $w_{2}$ to 2. Additionally, the values of $w_{1}$ and $w_{2}$ can be adjusted based on the specific requirements and characteristics of the driving scenario.

#### 4.1.2. Constraint Conditions

Discrete path point smoothing considers the physical constraints of the vehicle steering system and the positional constraints of the original obstacle avoidance path points.

Firstly, considering the vehicle steering system constraints, the vehicle driving along the smoothed path points should satisfy the constraint of the minimum turning radius $R_{min}$, assuming that three consecutive path points form a circle, as shown by the red dashed circle in Figure 10. Based on the principle of similar triangles, the turning radius corresponding to the current path point $p_{j}$ can be determined. Therefore, the turning radius at the current path point $p_{j}$ must satisfy the following constraint:

(26) $$\left|\vec{p_{j}p_{j-1}}+\vec{p_{j}p_{j+1}}\right|\leq \frac{{\left|\vec{p_{j}p_{j+1}}\right|}^{2}}{R_{min}}$$

To improve the computational efficiency in solving the path smoothness issue, the nonlinear constraint of the turning radius shown in Equation (26) needs to be linearized. Assuming that the distance between adjacent path points is approximately equal to the arc length between the two points, the expression $\left|\vec{p_{j}p_{j-1}}+\vec{p_{j}p_{j+1}}\right|$ can be Taylor-expanded, retaining only the first term to achieve linearization. By using this approach, the nonlinear turning radius constraint shown in Equation (26) can be transformed into the following linear constraint:

(27) $$F^{\prime }\left(X_{ref}\right)X\leq {\left(\frac{{\Delta s}^{2}}{R_{min}}\right)}^{2}-F\left(X_{ref}\right)+F^{\prime }\left(X_{ref}\right)X_{ref}$$

In the equation mentioned above, the expression for *F*(*X*) is determined as follows:

(28) $$F\left(X\right)={\left(x_{i-1}+x_{i+1}-{2x}_{i}\right)}^{2}+{\left(y_{i-1}+y_{i+1}-{2y}_{i}\right)}^{2}$$

(29) $$X={\left[x_{i-1},y_{i-1},{x_{i},y_{i},x}_{i+1},y_{i+1}\right]}^{T}$$

Furthermore, the smoothed waypoints should be positioned in the vicinity of the original waypoints. The expression for the position constraint is given below:

(30) $$\begin{array}{c} x_{oi}-X_{Buff}\leq x_{i}\leq x_{oi}+X_{Buff}\\ y_{oi}-Y_{Buff}\leq y_{i}\leq y_{oi}+Y_{Buff} \end{array}$$

where $X_{Buff}$ and $Y_{Buff}$ are positive threshold values, and their values increase as the road curvature increases. The values of $X_{Buff}$ and $Y_{Buff}$ represent the acceptable deviation from the desired trajectory and can be determined based on factors such as vehicle dimensions, safety margins, and local regulations. Additionally, the selection of these threshold values should be context-specific and may vary depending on the specific application and requirements.

In summary, the objective function COST and the two constraint conditions form the mathematical model of the discrete waypoint smoothing problem. By adopting the method described in [30], this smoothing problem can be transformed into a standard quadratic programming form for optimization and solution, resulting in a set of smoothed discrete waypoints.

### 4.2. Smooth Obstacle Avoidance Path Generation

To further enhance the smoothness of the obstacle avoidance path and achieve continuous changes in curvature, this paper employs piecewise fitting using fifth-degree polynomials to smooth the path points, thereby generating a smooth path. Assuming that the path generated with the DSF consists of *N*+1 waypoints, the obstacle avoidance path can be divided into *N* intervals. For each interval, two fifth-degree polynomials are used to represent the path. For instance, the representation of the *i*-th sub-interval polynomial is as follows:

(31) $$x_{r,i}\left(t\right)=\sum_{k=0}^{5}a_{k,i}t^{k}$$

(32) $$y_{r,i}\left(t\right)=\sum_{k=0}^{5}b_{k,i}t^{k}$$

In the equation mentioned above, the representation of the optimization variables for the *i*-th sub-interval is as follows:

(33) $$\begin{array}{c} A_{i}={\left[a_{0,i},a_{1,i},a_{2,i},a_{3,i},a_{4,i},a_{5,i}\right]}^{T}\\ B_{i}={\left[b_{0,i},b_{1,i},b_{2,i},b_{3,i},b_{4,i},b_{5,i}\right]}^{T} \end{array}$$

To solve the optimization problem and obtain the undetermined parameters $P_{i}=\left[A_{i};B_{i}\right]$ for the polynomial curves on each interval, we utilized a quadratic programming method for the solution. The objective function for optimization is defined as follows:

(34) $$cost=\sum_{i=1}^{N}\int_{t_{i-1}}^{t_{i}}\left[{\left({x_{r,i}}^{\left(3\right)}\left(t\right)\right)}^{2}+{\left({y_{r,i}}^{\left(3\right)}\left(t\right)\right)}^{2}\right]dt$$

where $t_{i-1}$ and $t_{i}$ represent the starting and ending time of each segment of the smooth sub-interval.

To ensure the continuity and smoothness of the final obstacle avoidance path formed by connecting the polynomial curves in each segment, several constraints need to be satisfied during the process of solving for the polynomial curve parameters. These constraints include the position, velocity, and acceleration constraints at the starting points and endpoints, as well as the position constraints for intermediate waypoints (excluding the starting points and endpoints). Furthermore, continuity constraints at the connection points of adjacent polynomial curves must be met. It is worth noting that all of these constraints are linear constraints.

## 5. Simulation Verification and Analysis of Results

### 5.1. Simulation Parameter Setting

In order to verify the performance of the proposed obstacle avoidance path-planning algorithm based on the driving safety field, the verification and comparative analysis were carried out based on the MATLAB 2021a platform, and the model parameters are shown in Table 1.

### 5.2. Simulation Result Analysis

To verify the feasibility and effectiveness of the path-planning method proposed in this paper for obstacle avoidance, three types of scenarios, namely, static obstacles, dynamic obstacles with low velocity, and multiple obstacles, were established, and the quality of the obstacle avoidance path generated using the proposed method was evaluated by comparing it with a path-planning method for obstacle avoidance based on the improved APF in [32].

#### 5.2.1. Static Obstacle Scene

**Figure 11:**
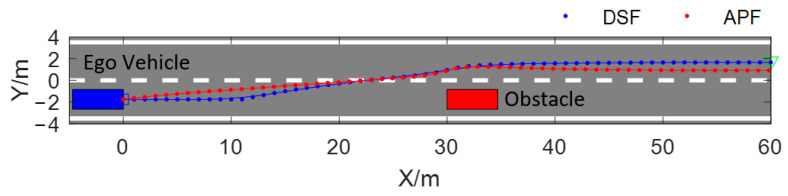
Comparison of obstacle avoidance path based on the DSF and the APF.

**Figure 12:**
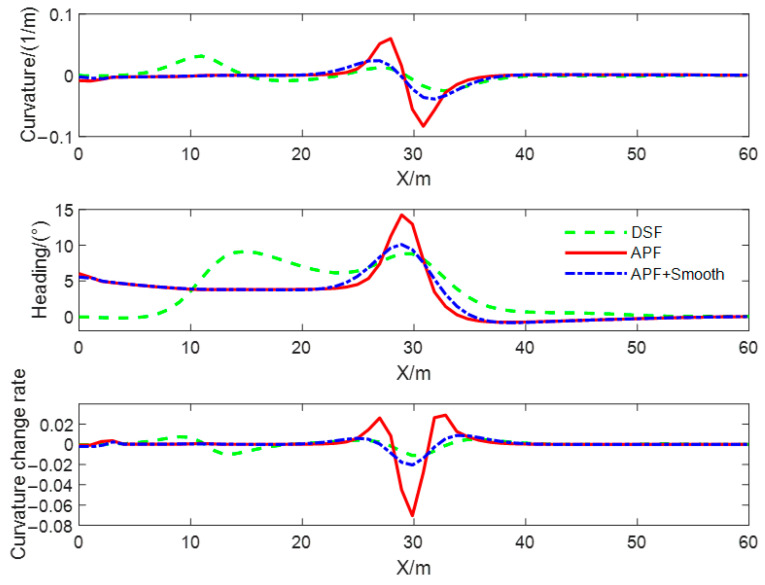
Comparison of curvature and heading angle in static obstacle scenarios.

**Figure 13:**
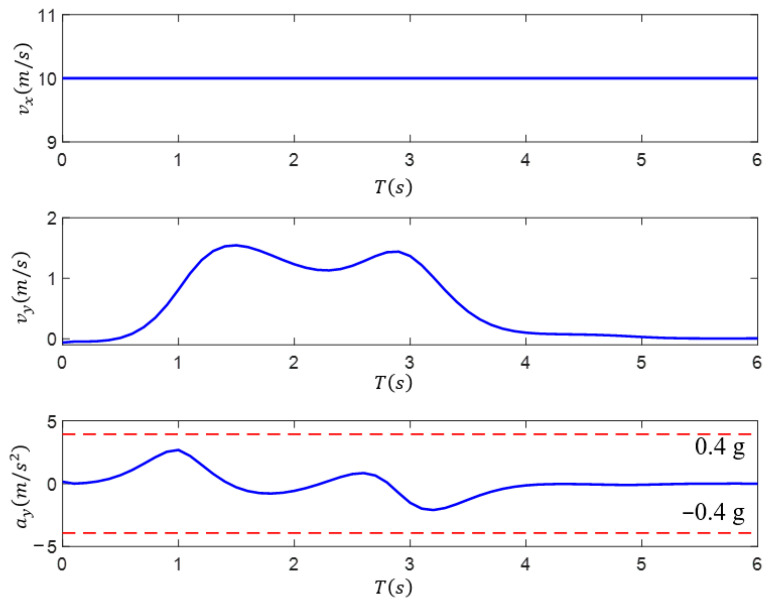
Vehicle velocity and lateral acceleration of the path based on the DSF.

**Figure 14:**
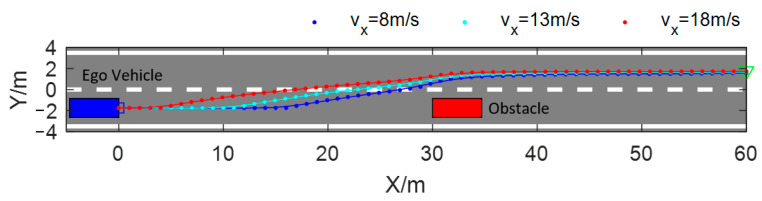
Path planning for obstacle avoidance at different velocities.

Test scenario 1 is a two-lane scenario with a static obstacle, as shown in Figure 11; the test vehicle is driving at a velocity of 10 m/s, the static obstacle is located in the lane where the test vehicle is located, and the test vehicle is required to complete the obstacle avoidance process by lane changing in a safe and smooth manner. The simulation results are shown in Figure 11, Figure 12, Figure 13 and Figure 14, respectively, showing the changes in curvature, curvature change rate, heading angle, lateral velocity, and lateral acceleration of obstacle avoidance paths generated with different algorithms.

Figure 11 shows the obstacle avoidance paths planned based on the DSF and the improved APF presented in [32]. The blue dotted line is the path planned based on the proposed method and the red dotted line is the path planned based on the improved APF. From Figure 11, it can be seen that the obstacle avoidance path planned based on the APF starts changing lanes earlier than the DSF, and the process of the obstacle avoidance path planned based on the APF is longer. Compared with the DSF, the test vehicle spends a longer time in a state of crossing two lanes, which increases the risk of collision. The path planned with the DSF is safer than the APF in this regard.

In order to compare the quality of the obstacle avoidance paths planned using different methods, and to verify the effectiveness of the smoothing method proposed in this paper, the obstacle avoidance paths are planned with the DSF, the APF, and the smoothed APF for the static obstacle scenario, and the curvature of the path, curvature change rate, and heading angle were, respectively, computed for the three types of generated obstacle avoidance paths, and the results of the computation are shown in Figure 12. The red solid line is the obstacle avoidance path based on the APF, the green dotted line is the obstacle avoidance path based on the DSF, and the blue dotted line is the obstacle avoidance path based on the APF with the smoothing method proposed in this paper, and we call this method APF + Smooth. From the comparison of curves, it can be seen that the curvature, heading angle, and curvature change rate of the obstacle avoidance path planned with the method proposed in this article are continuous and smooth. The maximum curvature of the obstacle avoidance path based on the DSF is the smallest and meets the minimum turning radius constraint. The maximum target turning angle is the smallest and meets the vehicle’s kinematics and dynamics constraints. The turning angle of the test vehicle at the initial and end times of lane changing is zero, which can ensure that the vehicle travels along the centerline of the lane. In addition, the minimum curvature change rate can provide high-quality control objectives for vehicle trajectory tracking and improve the riding comfort of drivers and passengers.

To quantitatively evaluate the quality of the obstacle avoidance paths, we selected four metrics for comparison: maximum curvature, maximum steering angle, distance to the target position, and minimum distance to obstacles (obstacle vehicles). These metrics are presented in Table 2. From these metrics, it can be inferred that the smaller the maximum curvature and the maximum steering angle, the better the maneuverability of the vehicle tracking obstacle avoidance path process, the closer the vehicle to the target position, and the higher the execution accuracy of the upper-level behavior decision results, ensuring that the vehicle accurately completes the predetermined driving task; in addition, the greater the minimum distance to the obstacle, the higher the safety of the vehicle in the obstacle avoidance process. From Table 2, it can be seen that the maximum curvature and the maximum steering angle of the obstacle avoidance path planned with the DSF are the smallest, which are reduced by 62.29% and 36.14%, respectively, compared with the improved APF. Even though the obstacle avoidance path generated with the improved APF algorithm adopts the smoothing algorithm proposed in this paper, the maximum curvature and the maximum steering angle are still slightly larger than those in the method proposed in this paper; the distance between the path endpoint and the target position for the obstacle avoidance path based on the DSF is less than 0.1 m. The minimum distance to the obstacle (obstacle vehicle) of the obstacle avoidance path based on the DSF is 2.72 m, which is better than that in the improved APF method, which verifies that the obstacle avoidance path generated with the DSF in the static obstacle scene is safer.

The DSF-based path-planning process assumes that the vehicle’s longitudinal velocity is kept constant, and then the lateral velocity and lateral acceleration can be obtained based on the calculation of the heading angle of the test vehicle as it drives along the planned path; moreover, the paths planned based on the driving safety field implicitly have information in the time dimension. Figure 13 shows the DSF-planned path corresponding to the lateral velocity and lateral acceleration change rule. From this figure, it can be seen that, during the obstacle avoidance operation before and after the end of the lane-changing maneuver, the lateral velocity is zero, the lateral acceleration shows continuous change, and the maximum value is less than 0.4 g. If the test vehicle’s path-tracking processes the implied time information as a control target, it can guarantee the stability of the test vehicle lane-changing obstacle avoidance operation.

The driving safety field constructed based on field theory has the characteristics of a vector field in physics, and the force that the test vehicle receives in the driving safety field is not only related to the field strength formed by the traffic elements but is also related to the motion state of the test vehicle. Whether the traditional or improved artificial potential field is used for obstacle avoidance path planning, the motion state of the test vehicle is not taken into account; therefore, faced with the scenario shown in Figure 11, regardless of the value of the auto-vehicle velocity taken, the trajectory planned with the APF is consistent with the red dotted line in Figure 11, and the planned path will not be able to adapt to the changes in the velocity of the test vehicle. Since the test vehicle is subjected to the field force in the driving safety field considering the test vehicle’s motion state, the problem can be effectively solved by planning the path using the method proposed in this paper. As shown in Figure 14, when the test vehicle passes through the same scenario shown in Figure 11 with different constant velocities, the obstacle avoidance path planned using the method proposed in this paper varies with the velocity of the test vehicle, and when the velocity of the vehicle is greater, the vehicle starts to change lanes at an earlier moment, i.e., farther away from the static obstacle, which allows enough longitudinal space to be reserved for lane changing. From the figure, it can be seen that when the vehicle velocity v_x_ = 10 m/s, the test vehicle starts to perform lane-changing obstacle avoidance at the position of x = 10 m, and when v_x_ = 18 m/s, the test vehicle starts to perform lane-changing obstacle avoidance at the position of x = 1 m. In order to illustrate the problem more clearly, Table 3 shows the positions of the two methods of planning obstacle avoidance paths across the target lane line for three different vehicle velocities. The position of the APF-planned path across the lane line is independent of the velocity of the ego vehicle, which is kept at x = 23.98. The method proposed in this paper has the ability to adapt to the state of the movement of the ego vehicle; with the rise in the velocity of the vehicle, the position of the vehicle across the lane line will be farther away from the obstacle vehicle, which can guarantee the safety of the vehicle obstacle avoidance process.

#### 5.2.2. Dynamic Obstacle Scene

**Figure 15:**
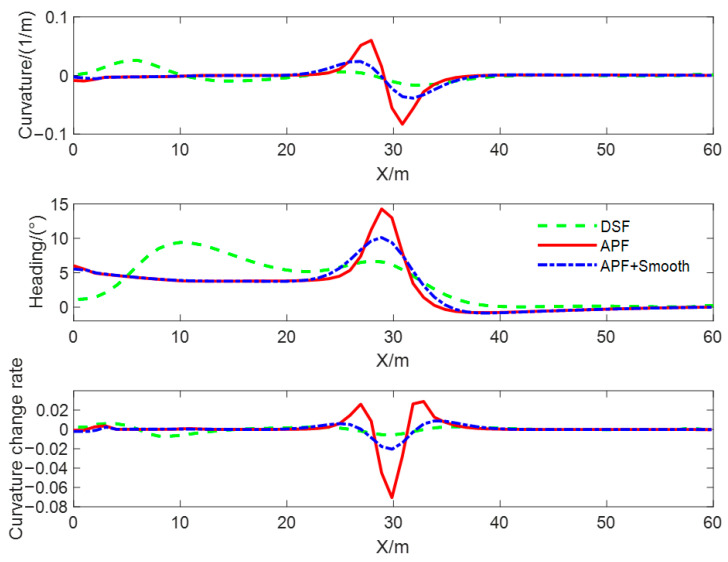
Comparison of curvature and heading angle in dynamic obstacle scenarios.

**Figure 16:**
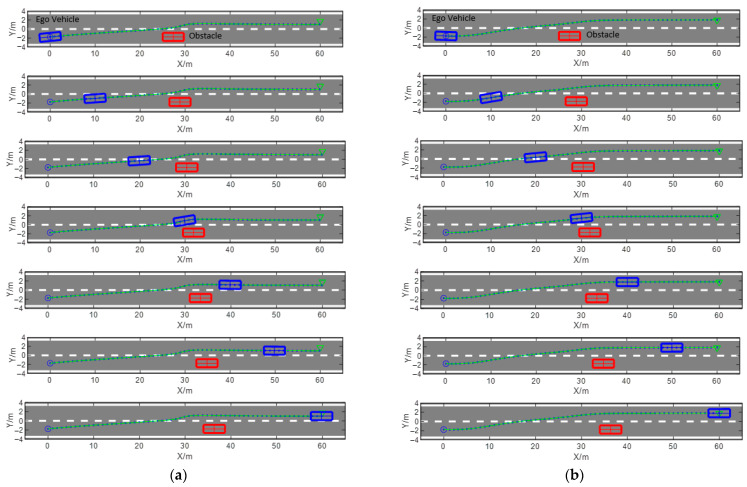
Obstacle avoidance path based on the APF and the DSF for dynamic obstacle scenarios: (**a**) path planning based on the APF; (**b**) path planning based on the DSF.

Test scenario 2 is a two-lane scenario with low-velocity obstacles; the test vehicle is driving at 10 m/s, and the low-velocity obstacle is located in the lane where the test vehicle is driving at 1.5 m/s. In order to improve driving efficiency, it is required that the test vehicle is able to complete the obstacle avoidance process by lane changing in a safe and smooth manner. The simulation results are shown in Figure 15 and Figure 16, which present the lane-changing and obstacle avoidance processes of the test vehicle tracking the planning path generated with the APF and the DSF, respectively. In Figure 16, the blue origin is the planning starting point, the green triangle is the planning endpoint, the blue box represents the test vehicle, the green box represents the low-velocity moving obstacle, and the green solid line represents the planned path, and the figure shows the relative positions of the test vehicle and the obstacle vehicle when it drives along the planned path with the velocity of 10 m/s to the position of $x=\left(0,10,20,30,40,50,60\right)$ in order from the top to the bottom. Comparing Figure 16a,b, it can be seen that at x=0, the heading angle of the test vehicle at the beginning of the path based on the APF is non-zero, and the test vehicle starts to perform lane changing; the heading angle at the beginning of the path based on the DSF is 0, and the test vehicle drives along the centerline of the current lane. At the position of x=30 m in the course of the lane-changing and avoidance process, the test vehicle driving along the planning path based on the DSF has already finished driving into the target lane, which decreases the possibility of collision with the obstacle vehicle, while the test vehicle driving along the planning path based on the APF is still in the position of crossing the two lanes, which increases the possibility of collision with the obstacle vehicle. At the end of the planning path, the planning path based on the DSF can realize the vehicle driving along the centerline of the target lane, while the planning path based on the APF has a certain deviation from the centerline of the target lane, which leads to the test vehicle possibly driving on the pressure of the lane line, which is not in line with the requirements of the traffic regulations and increases the driving risks. Comprehensive analysis shows that using the obstacle avoidance path-planning method proposed in this paper, the test vehicle can quickly complete the lane-changing process and ensure that driving is maintained along the centerline of the road at the planning starting point and the endpoint of the path. Due to the construction of the DSF-integrated consideration of the test vehicle, the lane line, and the impact of other traffic participants, compared with the APF, it can significantly improve driving safety.

A comparison of path curvature, curvature change rate, and heading angle of obstacle avoidance paths planned using different methods in dynamic obstacle scenarios is shown in Figure 15. From the comparison of curves, it can be seen that the curvature, heading angle, and change rate of the curvature of the obstacle avoidance path planned with the DSF in the dynamic obstacle avoidance scenario are continuous and smooth, and the steering angle of the test vehicle at the initial and the ending moment of the obstacle avoidance path based on the DSF is zero, which can ensure that the vehicle drives along the centerline of the lane and has the smallest rate of curvature change. Moreover, it can provide a high-quality target trajectory for lower trajectory tracking and improve the ride comfort of drivers and passengers. The steering angle of the path based on the APF is non-zero at the initial moment; the lane-changing behavior for obstacle avoidance starts earlier; the duration of the lane-changing process is longer; and the curvature, curvature change rate, and heading angle of the path change and obstacle avoidance process are larger than those of the method proposed in this paper.

Table 4 shows a comparison of the evaluation parameters of the obstacle avoidance paths under dynamic obstacle scenarios. From Table 4, it can be seen that the maximum curvature and maximum steering angle of the obstacle avoidance path planned with the DSF are the smallest, which are reduced by 68.95% and 34.11%, respectively, compared with the improved APF. Even though the obstacle avoidance path planned with the improved APF algorithm was smoothed using the smoothing algorithm proposed in this paper, the maximum curvature and maximum steering angle are still slightly larger than the proposed method. The distance between the path endpoint and the target position for the obstacle avoidance path based on the DSF is less than 0.1 m; therefore, the obstacle avoidance path can guide the test vehicle to reach the target position smoothly; the obstacle avoidance path based on the DSF has the largest minimum distance to the obstacle (obstacle vehicle) and is larger than that generated in the static obstacle scenarios, which verifies that the obstacle avoidance path generated with the DSF in the dynamic obstacle scenarios is safer.

#### 5.2.3. Multiple Obstacle Scene

**Figure 17:**
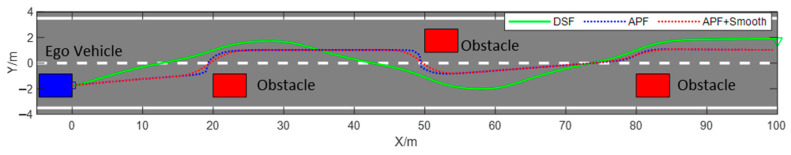
Obstacle avoidance path planning in multiple obstacle scenarios.

Test scenario 3 is a two-lane scenario with multiple obstacles; the test vehicle is driving at a velocity of 10 m/s; and there are three obstacle vehicles on the road ahead, which requires the test vehicle to complete the obstacle avoidance process in a safe and smooth manner. The obstacle avoidance paths in multiple obstacle scenarios are shown in Figure 17, where the green solid line is the multiple obstacle avoidance path generated with the DSF, the blue dashed line is the obstacle avoidance path generated using the improved APF, and the red dashed line represents the obstacle avoidance path generated with the improved APF after smoothing. Comparing the obstacle avoidance paths generated using the different methods in this scenario, it can be seen that the quality of the obstacle avoidance path generated with the improved APF is improved after smoothing, but the obstacle avoidance path point is still too close to the obstacle, increasing the risk of the obstacle avoidance process. Moreover, after the obstacle avoidance behavior is completed, the distance between the path endpoint and the target position is relatively far. In contrast, the obstacle avoidance path generated with the DSF maintains a safe distance from the obstacle vehicle, which can improve the safety of the obstacle avoidance process. After completing the obstacle avoidance operation, the test vehicle can smoothly reach the target position.

**Figure 18:**
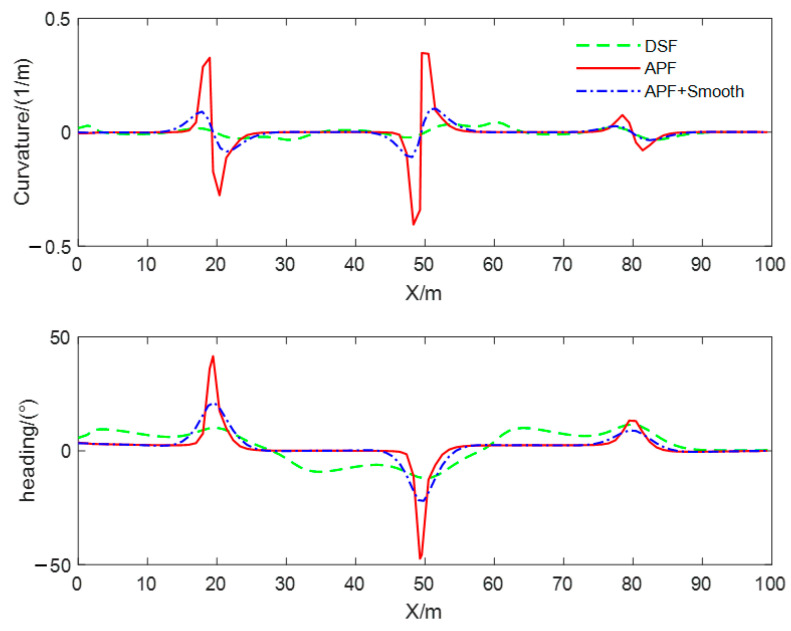
Comparison of curvature and heading angle in multiple obstacle scenarios.

The curves of the curvature and heading angle of the obstacle avoidance path generated using three methods in multiple obstacle scenarios are shown in Figure 18. From this figure, it can be seen that the maximum curvature of the obstacle avoidance path generated with the improved APF is close to 0.5 $m^{-1}$, and the maximum heading angle is close to 50°. After smoothing, the curvature becomes smoother and continuous, and the maximum heading angle decreases by more than 50%. This verifies that the proposed smoothing method is suitable for smoothing discrete obstacle avoidance waypoints. In contrast, the obstacle avoidance path generated with the DSF is smooth and continuous, with small changes in curvature and heading angle. This verifies that the obstacle avoidance path generated using the proposed method in multiple obstacle scenes has higher quality and can reduce the difficulty of trajectory tracking control.

The evaluation parameters of the obstacle avoidance paths generated using the three methods in the multiple obstacle scenario are shown in Table 5. From Table 5, it can be seen that the maximum curvature and the maximum steering angle of the obstacle avoidance path planned with the DSF are the smallest, which are reduced by 87.32% and 72.06%, respectively, compared with the improved APF. Even though the obstacle avoidance path planned with the improved APF algorithm was smoothed using the smoothing algorithm proposed in this paper, the maximum curvature and maximum steering angle are still slightly larger than the proposed method. The distance between the path endpoint and the target position for the obstacle avoidance path based on the DSF is less than 0.2 m, and the obstacle avoidance process can guide the test vehicle to reach the target position smoothly; the minimum distance to the obstacle (obstacle vehicle) of the obstacle avoidance path planned with the DSF is 2.72 m, while the minimum distance to the obstacle vehicle of the obstacle avoidance path planned with the improved APF is less than 1.5 m, which verifies that the obstacle avoidance path generated using the proposed method in the multiple obstacle scenario is safer.

## 6. Conclusions

In response to the issues of the curvature-discontinuous path and the lack of consideration for the influence of the vehicle’s motion state in the application of improved artificial potential field (APF) to obstacle avoidance path planning, a dynamic path-planning method for obstacle avoidance based on the driving safety field (DSF) is proposed in this work. The method decomposes the path planning for obstacle avoidance into two steps. Firstly, the driving safety field theory is utilized to establish a comprehensive driving safety field for the obstacle avoidance scenario. By employing a virtual kinematic system, a collision-free feasible lane-changing path is generated. Secondly, the obstacle avoidance path is smoothed using quadratic programming, resulting in a dynamically continuous and smooth obstacle avoidance path. Simulation and comparative analyses were conducted in scenarios with static or dynamic single and multiple continuous obstacles. Compared with the improved APF, the path-planning algorithm for obstacle avoidance proposed in this paper generates a collision-free and curvature-continuous path, significantly reducing the maximum curvature, maximum heading angle, and distance to the target position of the obstacle avoidance path. Moreover, the minimum distance to the obstacles is significantly better than that of the comparative methods. Additionally, the proposed method automatically adjusts the starting position of the obstacle avoidance maneuver based on the vehicle’s motion state. As the relative velocity between the ego vehicle and the obstacle vehicle increases, the starting position of the obstacle avoidance path is set farther away from the obstacle vehicle, allowing for sufficient longitudinal space, which improves the safety of the lane-changing process. Therefore, the proposed method satisfies the requirements of obstacle avoidance safety, comfort, and stability for intelligent vehicles in complex environments. In order to enhance its applicability, future work involves expanding this method to scenarios that encompass complex factors such as pedestrians, vehicles, and road conditions. By deploying and testing the algorithm in real vehicles, further improvements in algorithm performance can be achieved.

## Figures and Tables

**Table 1:** Main parameter settings for simulation.

Symbol	Description	Value	Unit
$R_{a},R_{b},R_{c},R_{j}$	Road condition impact factor	1	--
$D_{t}$	Maximum influence distance of the potential field	30	m
${LT}_{a}$	Lane line type parameter	1	--
K	Potential field adjustment coefficient	1	--
$k_{1}$	Lane line distance coefficient	2	--
$k_{2}$	Driving safety field distance coefficient	3	--
$k_{3}$	Driving safety field velocity coefficient	0.03	--
$k_{D}$	Driving field adjustment coefficient	100	--
$k_{if}$	Adjustment coefficient for car following behavior	0.15	--
$k_{ic}$	Adjustment coefficient for lane-changing behavior	0.20	--
$L_{w}$	Lane width	3.5	m
$w_{1}$	Coefficient of smoothness objective function	5	
$w_{2}$	Coefficient of similarity objective function	2	
L	Wheelbase of the vehicle	2.80	m
$L_{f}$	Distance from the front axle to the vehicle’s forefront	0.96	m
$L_{r}$	Distance from the rear axle to the vehicle’s rearmost	0.92	m
$L_{b}$	Width of the vehicle	1.94	m

**Table 2:** Comparison of obstacle avoidance path parameters in static obstacle scenarios.

	Maximum Curvature (1/m)	Maximum Heading Angle (°)	Distance to Target Position (m)	Minimum Distance to Obstacles (m)
DSF	0.03	9.11	0.08	2.72
APF (Baseline)	0.08	14.26	0.74	2.63
APF + Smooth	0.04	10.11	0.74	2.58
Comparison	62.29%	36.14%	89.12%	3.43%

**Table 3:** Comparison of obstacle avoidance paths at different velocities.

Position Across Lane Line	$v_{x}\;=\;8\;m/s$	$v_{x}\;=\;13\;m/s$	$v_{x}\;=\;18\;m/s$
DSF	x = 26.85	x = 22.46	x = 16.72
APF	x = 23.98	x = 23.98	x = 23.98

**Table 4:** Comparison of obstacle avoidance path parameters in dynamic obstacle scenarios.

Methods	Maximum Curvature (1/m)	Maximum Heading Angle (°)	Distance to Target Position (m)	Minimum Distance to Obstacles (m)
DSF	0.03	9.40	0.07	3.14
APF (Baseline)	0.08	14.26	0.74	2.63
APF + Smooth	0.04	10.11	0.74	2.58
Comparison	68.95%	34.11%	90.85%	19.31%

**Table 5:** Comparison of path parameters in multiple obstacle scenarios.

Methods	Maximum Curvature (1/m)	Maximum Heading Angle (°)	Distance to Target Position (m)	Minimum Distance to Obstacles (m)
DSF	0.04	11.62	0.15	2.72
APF (Baseline)	0.35	41.58	0.95	1.33
APF + Smooth	0.10	21.18	0.95	1.74
Comparison	87.32%	72.06%	83.76%	104.90%

## Data Availability

Data are contained within the article.
